# New perspectives on substituted relational autonomy for shared decision-making in critical care

**DOI:** 10.1186/s13054-018-2187-6

**Published:** 2018-10-11

**Authors:** Nicola Grignoli, Valentina Di Bernardo, Roberto Malacrida

**Affiliations:** 1 Sasso Corbaro Medical Humanities Foundation, Via Lugano 4b, CH-6500 Bellinzona, Switzerland; 2 Clinical Ethics Commission, Ente Ospedaliero Cantonale, CH-6500 Bellinzona, Switzerland; 3 Psychiatry Consultation Liaison Service, Organizzazione Sociopsichiatrica Cantonale, CH-6850 Mendrisio, Switzerland; 4 Intensive Care Unit, Ospedale Regionale di Lugano, Ente Ospedaliero Cantonale, CH-6900 Lugano, Switzerland

**Keywords:** Critical care, Medical ethics, Psychology, Shared decision-making, Relatives

## Abstract

In critical care when unconscious patients are assisted by machines, humanity is mainly ensured by respect for autonomy, realised through advance directives or, mostly, reconstructed by cooperation with relatives. Whereas patient-centred approaches are widely discussed and fostered, managing communication in complex, especially end-of-life, situations in open intensive care units is still a point of debate and a possible source of conflict and moral distress. In particular, healthcare teams are often sceptical about the growing role of families in shared decision-making and their ability to represent patients’ preferences. New perspectives on substituted relational autonomy are needed for overcoming this climate of suspicion and are discussed through recent literature in the field of medical ethics.

## Context and background

One of the principal challenges for the future of critical care is how to ensure respect and dignity: while unconscious patients are being assisted by machines, their humanity is mainly preserved by consideration of their individual will, values and priorities [1]. The principle of autonomy—defined in clinical practice as the right to freely make informed choices—is the cornerstone of contemporary medical ethics and will probably be a constant moral value in the coming decades. Its application has long been a topic of debate in bioethics [2] and has been a focus of recent discussions related to the need for shared decision-making (SDM) in critical care [3–5].

Up to 95% of critically ill adults are unable to make autonomous choices [6] and SDM is a necessary and long-standing reality in intensive care units (ICUs). In accordance with international guidelines [7] and the laws in force in many European countries [8], the will of incapacitated patients in SDM can be reconstructed in two ways: (1) implementation of advance directives and (2) cooperation with relatives, who may be “partners in the decision” or legal surrogates. Cooperation seems to be the most widespread solution [9] because, prior to being implemented, advance directives frequently need to be interpreted through an in-depth discussion between clinicians and patients’ families [10]. That, however, is a source of major conflicts in communication and of moral distress [11].

Recent acquisitions in bioethics are potentially of great help for practitioners in ICUs confronted daily with complex moral issues: at least one end-of-life decision precedes the majority of deaths in North American and European ICUs [12]. In ICUs concepts of bioethics evolve more slowly than biotechnology. They should be regularly refreshed and their relevance permanently questioned: do we need a new conceptual framework on substituted autonomy for SDM in critical care?

## Autonomy in critical state

In recent years the primacy of autonomy has seen its progressive extension from the purely decisional context of informed consent to the doctor–patient relationship. It is spreading as a new standard in health communication, reversing traditional medical paternalism and empowering the patient. Patient-centred approaches clearly identify the exercise of autonomy with self-determination and competence to consent, thus being exposed to the risk of confusing the right to a proper process of deliberation with a good moral choice. For some, autonomy is more than a right: making one’s own choice is seen as an intrinsic value [13], undermining the role of other ethical principles. Besides these traditional points of view, alternative approaches in bioethics seem to better encompass the progressive evolution of the physician–patient relationship over recent years, in which the dual communication model has been extended to ICU teams of healthcare professionals until becoming a more fluid “care-cooperative” approach [14]. Faced with relatives, physicians tend to quit prescriptive roles, thus becoming facilitators of the decision-making process or assuming a collaborative role [15]. In particular, relational models of autonomy inspired by phenomenology and feminist perspectives [16, 17] answer the current need to reconstruct preferences through comparing and integrating different opinions: from this point of view deliberation is not purely selfish but is shared, responding to the characteristics that future generations will bring to critical care. In particular, members of the “millennium generation” are now permanently connected with each other through social media; moreover, they are informed in a shared and cumulative way [18] that tends to develop as a sort of “community-based informed consent”.

New conceptions of relational autonomy seem to better illustrate what is actually done in the decision-making process in ICUs: a new ethical perspective founded on a collaborative model has emerged [5]. Achieving awareness of this new perspective and integrating it in the everyday practice of SDM [17] could represent a challenge—a chance for bridging the gap generated by the evolution of communication in critical care while respecting the principle of autonomy as a human value in an even more technological environment [1]. SDM is in fact an internationally recognized solution for reconstructing the will of critically ill patients; opinions derived from clinical practice, however, tend to be discordant.

## Seize the challenge of SDM

Relatives (by which we here also mean partners and close friends) share significant characteristics with patients relating to their personal sphere and there is widespread consensus on the importance of their major involvement in SDM processes. Despite these facts, clinicians remain sceptical about the role of relatives in representing patients’ preferences. Studies suggest that conflicts between healthcare providers and patients’ families occur in nearly two-thirds of cases, and decisions at the end of life are indeed indicated as a major source of conflicts in ICUs [11]. Potential consequences of such conflicts are of primary interest. A growing body of evidence shows that terminal care, the perception of futile treatment (especially if at the insistence of patients’ family members) and disagreements about treatment at the end of life are all important drivers of moral distress and burnout [19]. At the same time, family members called to act as partners in decision-making processes are exposed to a burden that can be potentially harmful. The risk of developing psychological disorders that might impact their future quality of life are now well documented [20].

These different sources of difficulty can create barriers to implementing the real possibility of positive cooperation with relatives. Misunderstandings in communication and the belief that involving families in decision-making may be harmful for both sides create a climate of suspicion that should be carefully avoided. SDM remains, therefore, a challenge for ICUs [21]. The absence of any prior relationship between clinicians and patients and/or their relatives and the need to make difficult and sometimes urgent decisions play an important role when confronted with the need to build an effective collaboration. Besides, recent studies and debates have focused on a plethora of aspects of SDM that can raise difficult issues. These range from the definition of SDM to the discussion of concepts such as futile and potentially inappropriate treatment [22] or debate about the way to involve family members [23]. Among the various proposed solutions to cope with the difficulties of SDM in ICUs, the ability of clinicians to establish and maintain a good relationship and effective communication with relatives appears crucial but difficult to promote. What is new in this regard and what are the most important challenges and solutions (Table 1)?

**Table 1:** Challenges and solutions for granting autonomy in open ICUs

Challenges	Solutions
• Balancing ethical responsibilities in SDM	• Cooperation • Relational autonomy
• Preventing burnout	• Consultant psychologist
• Preventing moral distress	• Ethical advice
• Managing interpersonal conflicts in end-of-life SDM	• Interdisciplinary meetings • Staff management and dispositional organization
• Providing valuable information	• Structured communication tools
• Preventing relatives’ psychological disorders	• Physician's social, psychological and ethical skills • Training
• Embedded accuracy of the relatives’ predictions	• Offering support to relatives

First of all, SDM is defined as a collaborative process that allows patients, or their relatives, and clinicians to make therapeutic decisions together, by taking into account the best scientific knowledge as well as the available understanding of the patient’s preferences [22, 24]. At the same time, most family members prefer to be involved in a process of cooperation rather than maintain a high degree of control over decisions or, conversely, they leave that control to clinicians [25]. Furthermore, in order to share information effectively, evidence [26] suggests arranging a meeting with families as soon as possible after the patient’s admission to an ICU, scheduling regular meetings and involving members of the interdisciplinary team (physicians, nurses, therapists) in discussions whenever changes in the clinical situation arise. Through this process structured communication tools may be supportive to collect and to trace data on decision-making [26]. Finally, evidence of the psychological effects on relatives involved in treatment choices in end-of-life care shows that support offered by clinicians is a key element in containing stress and negative feelings and in preventing the development of a sense of guilt [20].

Implementing SDM as a tool for granting autonomy requires specific investment in staff management, dispositional organization and healthcare professionals’ training. For achieving these goals, creating “open” ICUs is a major cultural change [27].

## “Open” ICUs: still on the way

Opening ICUs to families by liberalizing visiting policies is clearly identified as the recommended prerequisite for establishing and maintaining good communication [26]. In short, opening up ICUs improves cooperation, facilitating better protection of the patient’s right to autonomy and increasing the quality of care [21]. Nevertheless, empirical data [28] show that there is still widespread scepticism among clinicians about the benefits of the presence of family members in ICUs. Although SDM is widely adopted in clinical practice [4], doubts still remain about the ability of relatives to become critical partners in decision-making. Two main core difficulties can be summarized: (1) understanding relevant medical information and appreciating consequences; (2) being morally responsible for the wishes of the patient. Obviously, medical communication in critical care might not be well understood by family members but data also show that clinicians’ social skills have an impact on their perception of the prognosis [21]. Furthermore, accuracy of the relatives’ predictions regarding the patient’s wishes seems to be low [29]. Due to these difficulties, not surprisingly, in both Western Europe and in the United States, many ICUs continue to adopt restrictive visiting policies [30]. Recently more objective (statistical) tools have been proposed to ensure better compliance with the patient’s wishes [31]. While awaiting further development of these tools and considering that the role of relatives is—in several countries—laid down by law, our attention should necessarily focus on critical issues related to their involvement in SDM (Table 2).

**Table 2:** Present difficulties and future opportunities for relatives involved in SDM in open ICUs

Difficulties	Opportunities
• Understanding medical information ○ Appreciating consequences ○ Relevance accorded to technical and clinical information ○ Emotional context	• Involvement in regular meetings ○ Collect and trace relevant data on the basis of clinical changes ○ Prevent and detect misunderstandings ○ Benefit from regular and effective communication
• Assuming moral responsibility for the wishes of the patient ○ Defining relatives’ personal values and priorities ○ Defining power of attorney (conflicts within relatives)	• Provide patient’s personal information ○ Perceived health-related quality of life ○ Character and will to live (demonstrated resilience) ○ History of illness
• Low accuracy in predicting patient’s preferences • Disagreements about goals of care (especially in end-of-life situations)	• Share responsibilities ○ Possibility to engage with the interdisciplinary team ○ Possibility to check other sources of information (web, social media)
• Exposure to emotional burden and psychological disorders	• Maintain and share intimacy with patient

It is necessary, therefore, to highlight some significant points which show that open ICUs can be, at least for now, an opportunity to make choices that are more in conformity with the interests of patients who are incapable of discernment. In recent research [32], aspects related to the presence of relatives were explored using the term “intimacy”, meaning a personal relationship based on shared feelings and emotions and including elements such as values that are more involved in decision-making. Results from this study reveal that families share significant characteristics with patients related to their personal sphere, but clinicians do not recognise this and are critical. In line with these results, other data show that clinicians seem to underestimate the contribution of other factors considered crucial by relatives in their decision-making, such as their knowledge of the patient’s strength of character and will to live, the history of illness and resilience demonstrated by the patient, observation of the patient’s appearance and the belief that their presence can have a positive influence on the prognosis [29, 33].

## “Substituted relational autonomy”

Some intertwined ethical concepts that can helpfully clarify and develop the concept of autonomy in ICUs should at this point be addressed (Table 3).

**Table 3:** Main characteristics of traditional and relational models of autonomy

Traditional model	Relational model
• Freedom of choice	• Freedom of choice
• Primacy of informed consent	• Role and influence of others on expanding individual’s ability to make choices
• Voluntariness • Independence • Self-sufficiency	• Consider influence of relatives that is wanted and expected by some patients
• Maintenance of control over situations	• Health and sickness viewed also as interpersonal and family events
• Ability to exercise proper responsibility	• Role of social relationships in developing individual’s identity
• Avoidance of interference and undue pressures	• Adapt individual preferences to the needs of loved ones
• Clear boundaries between self and others	• Importance of personal relationships and shared interests

The strictly individualistic theory on autonomy assumes that this principle is based on the concern, dominant in bioethical theory and clinical practice, to protect the individual’s freedom of choice from unwanted interference in their decisions [16]. Autonomy coincides with the duty to guarantee free, voluntary and informed consent for the individual: information and non-interference seem not only necessary, but also sufficient conditions for ensuring a free choice. The duty of non-interference is also extended to relatives and can contribute to clinicians’ scepticism about SDM [34]. At times, even competent patients who decide to delegate their wishes to others or to give great importance to the needs and wishes of their loved ones are considered subject to undue pressure or unable to exercise their responsibilities fully [35]. Therefore, from this perspective of autonomy based on values of independence, control and self-sufficiency, individuals are considered separated from others by boundaries that can only be crossed by voluntary consent [36].

On the contrary, the relational theory of autonomy [16, 17] recognises the inherent meaning of personal relationships, characterised by intimacy, community, peculiarity, non-consensuality, sensitivity and favouritism. It recognizes the essential role of social relationships in the make-up of the individual’s identity and ability to make decisions. It assumes that choices concerning health and sickness are hardly ever solely a personal problem for the patient but often involve others, thus becoming interpersonal and family events. Many patients welcome and expect the influence of relatives; they adapt their preferences to meet the needs and wishes of those whose interests are shared [37].

Understanding autonomy as a relational process seems an opportunity for overcoming a climate of suspicion regarding the patient’s family, considering the latter as part of a new kind of relationship of trust that can ensure that the patient’s wishes are respected [38].

**Fig. 1:**
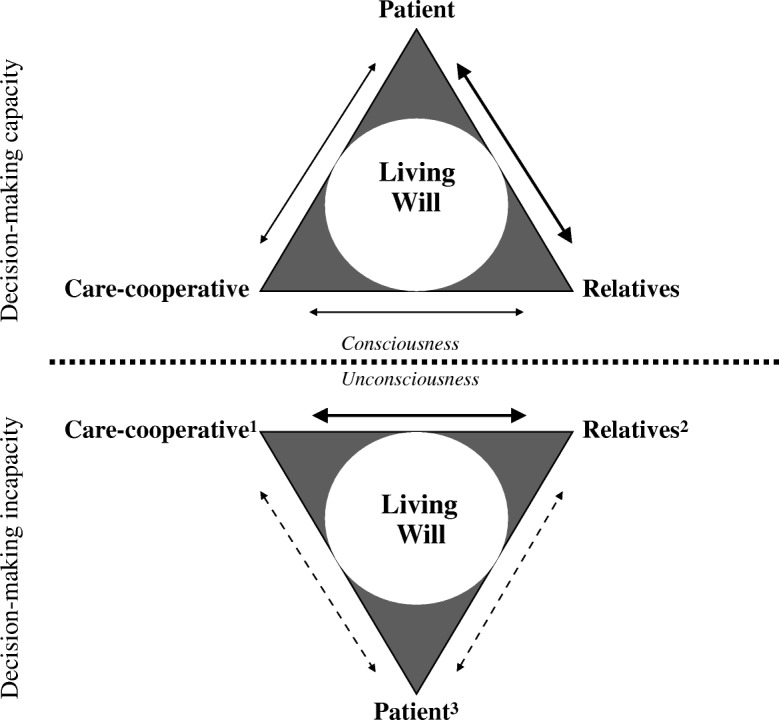
The substitute relational autonomy model for SDM in critical care. *1* Previous discussion with patients of therapeutic procedures, clinical team-shared opinion. *2* Knowledge of patients’ health-related quality of life, character and will to live (demonstrated resilience), history of illness. *3* Advanced directives, previous opinions, non-verbal communication. Narrowing represents communication links between parties involved in SDM in critical care

**Fig. 2:**
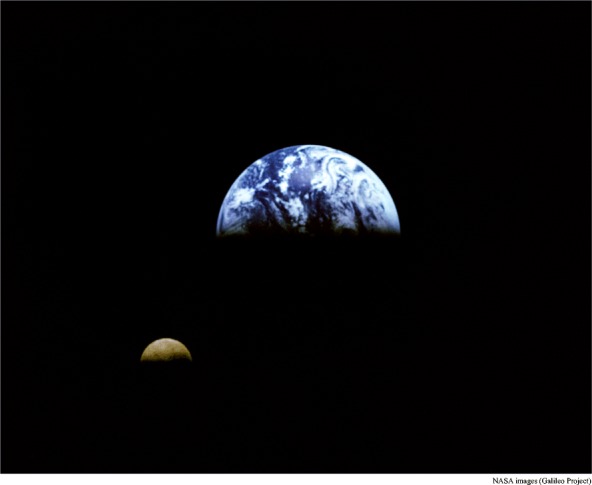
Moonlight as an illustration of substituted relational autonomy in critical care. Taking the earth as the individual and its satellite the moon as the relative, moonlight can be seen as what can still shed light on a living will during the night of an unconscious state

Trust allows part of the decision-making process to be delegated to others, in a way that can be seen as an indispensable condition for the exercise of autonomy and a valuable opportunity for sharing responsibility in SDM [3, 39]. What can be defined as “substituted relational autonomy” speaks to all parties involved in decision-making processes (Figs. 1 and 2) and its reciprocity leads to the need to evaluate it in an interdependent manner: trust can be defined in terms of favourable expectations regarding respect for the rights of others.

This is exactly what is needed in SDM in critical care. Understanding autonomy relationally and its potential impact on a relationship of trust [14] can represent a shift in a needed psychological defence mechanism in ICUs, from avoidance to openness to others, from individualism to mutual support and cooperation. Through trusting the patient’s relatives, clinicians will in turn be trusted and so hopefully increase their level of compassion satisfaction, a crucial positive factor linked with professional quality of life in ICUs [19].

## Limits and pitfalls

Obviously, relationships with the family may not necessarily be positive (and sometimes, especially, an incapacitated patient should be appropriately protected in this sense). Pitfalls can present themselves in different forms: passing from difficulty to acknowledge moral responsibility for the wishes of the patient (in terms of sense of guilt [20] or accuracy [29]), to uncertainty and the need to find the right balance over who in fact has the right to become a partner in the SDM process, up to the attempt to pursue egoistic interests in order to avoid family conflicts [40]. Bearing in mind these limits, even the awareness of the existence of conflictual relationships, actual or supposed, between the patient and relatives may be useful in ICUs to give full meaning to the concept of autonomy by shedding light on family relationships.

## Conclusions

In a clinical environment where patients are mostly unconscious and assisted by machines, where clinicians are a community of care and where relatives are increasingly connected through new technologies and social media, respect for the individual’s will can be considered a major challenge. Autonomy of choice, especially of those who are incapable of discernment, can be seen as a relational faculty jointly constructed that could contribute to maintain and foster humanisation in critical care. To achieve this goal, the psychological and ethical skills of the ICU team need to be improved by specific training programmes and in critical situations, liaison with a consultant psychologist or ethical advice could be helpful. New perspectives offered by substituted relational autonomy seem to offer new educational possibilities for implementing a sustainable practice of SDM in ICUs.

## Abbreviations

ICU: Intensive care unit
SDM: Shared decision-making
